# Structure and function of voltage-gated sodium channel Nav1.6: Involvement in the pathological process of neural injury

**DOI:** 10.4103/NRR.NRR-D-25-00354

**Published:** 2025-09-29

**Authors:** Huaiyuan Wang, Yuhang Wei, Junqi Wang, Jiyuan Liu, Shaowu Ou, Jun Wang

**Affiliations:** Department of Neurosurgery, The First Hospital of China Medical University, Shenyang, Liaoning Province, China

**Keywords:** Alzheimer’s disease, amyotrophic lateral sclerosis, brain injuries, epilepsy, multiple sclerosis, Nav1.6, nervous system diseases, neural regeneration, Parkinson’s disease, voltage-gated sodium channel

## Abstract

The voltage-gated sodium channel Nav1.6, encoded by the sodium voltage-gated channel alpha subunit 8 gene, is a crucial regulator of neuronal excitability, with widespread expression throughout the central and peripheral nervous systems. Recent breakthroughs in structural biology, particularly the elucidation of the cryo-EM architecture of Nav1.6 at a resolution of 0.31 nm, have provided unprecedented insights into its molecular organization and functional modulation. As a key mediator of action potential initiation and propagation, Nav1.6 possesses unique biophysical properties, including persistent and resurgent sodium currents that critically influence neuronal firing patterns. This comprehensive review synthesizes current knowledge on the physiological functions and pathological roles of Nav1.6 in multiple neurological conditions. Key findings include the following: (1) Epilepsy studies reveal more than 250 sodium voltage-gated channel alpha subunit 8 mutations with distinct genotype–phenotype correlations, where gain-of-function variants lead to severe epileptic encephalopathies, while loss-of-function variants are associated with generalized epilepsy, highlighting the potential of Nav1.6-selective blockers such as XEN901 and GS967. (2) In Alzheimer’s disease, Nav1.6 mediates amyloid-β oligomer-induced neuronal hyperexcitability through amyloid precursor protein-dependent membrane trafficking and regulates beta-secretase 1 expression via nuclear factor of activated T cells 1 signaling, suggesting novel disease-modifying strategies. (3) Parkinson’s disease research has demonstrated that Nav1.6 upregulation in reactive astrocytes in the globus pallidus contributes to motor deficits through calcium-mediated abnormalities in neuronal synchronization. (4) Amyotrophic lateral sclerosis involves Nav1.6-dependent cortical hyperexcitability preceding motor neuron degeneration, with riluzole showing partial efficacy through sodium current modulation. (5) Multiple sclerosis pathophysiology features Nav1.6 redistribution in demyelinated axons, which drives calcium-dependent axonal injury via reverse Na^+^/Ca^2+^ exchange. (6) Chronic pain mechanisms involve Nav1.6 overexpression in dorsal root ganglia neurons, regulated by the p38 mitogen-activated protein kinase and tumor necrosis factor-α signaling pathways. (7) Traumatic brain injury models show that exercise-induced cognitive improvement is correlated with the normalization of Nav1.6-mediated excitability. Therapeutic development has progressed from nonselective sodium channel blockers to precision approaches, including state-dependent pore blockers designed using structural insights; allosteric modulators targeting specific conformations; gene therapy strategies using clustered regularly interspaced short palindromic repeats and antisense oligonucleotides; and miRNA-based regulation of channel expression. Current challenges include achieving sufficient subtype selectivity, optimizing blood–brain barrier penetration, and developing clinically relevant biomarkers for patient stratification. Future directions emphasize the integration of advanced technologies—such as single-cell multiomics to map neuronal subtype-specific expression patterns, patient-derived organoids for personalized drug testing, and machine learning-assisted drug design—to accelerate translation. Large-scale collaborative efforts will be essential to validate therapeutic candidates and establish genotype-guided treatment protocols for Nav1.6-related disorders.

## Introduction

**Figure 1:**
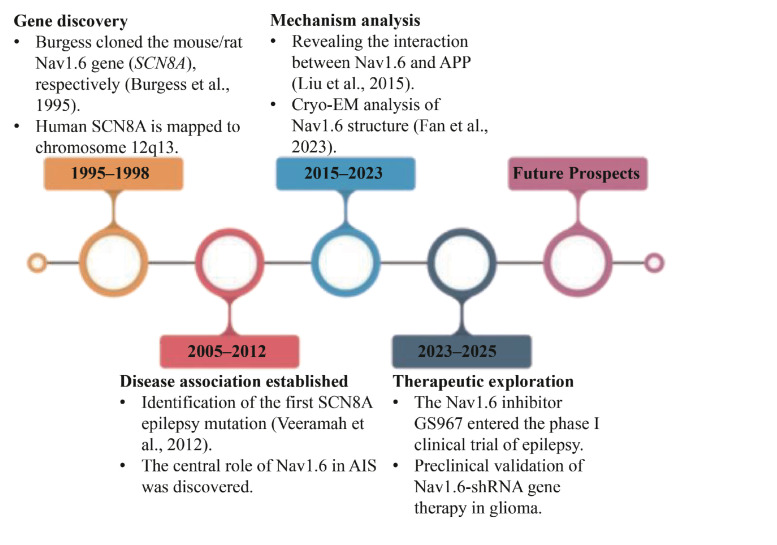
Key milestones in Nav1.6 research. More than 30 years have passed since the first discovery of SCN8A. Research on Nav1.6 has focused on its structure, expression, function, and electrophysiology. With increasing in-depth research, the application of Nav1.6 will be more extensive in the future. AIS: Axon initial segment; APP: amyloid precursor protein; cryo-EM: cryo-electron microscopy; SCN8A: sodium voltage-gated channel alpha subunit 8; shRNA: short hairpin RNA.

Voltage-gated sodium channels (VGSCs) are a class of macromolecular transmembrane proteins that are widely distributed on the surface of cell membranes (Catterall, 2017; Wood and Iseppon, 2018; Eshed-Eisenbach and Peles, 2021; Huang et al., 2024). Nine subtypes of VGSCs are known, including Nav1.1–Nav1.9, which are critical drivers of excitability in excitable cells (Catterall, 2017, 2023; Wang et al., 2017; Wood and Iseppon, 2018; Catterall et al., 2020; Eshed-Eisenbach and Peles, 2021). Among them, Nav1.6 is a subtype of VGSC that is widely distributed in the nervous system. Since it plays an important role in the generation and conduction of action potentials (APs) in neurons, it is considered a key ion channel or initiator ion channel for neuronal excitability (Hu et al., 2009; Ye et al., 2018; Sole and Tamkun, 2020; Zybura et al., 2021; Li et al., 2023; **Figure 1**).

Research has shown that Nav1.6 not only plays a key role in the normal electrophysiological activities of neurons but is also closely related to the occurrence and development of various nervous system diseases, such as epilepsy, Alzheimer’s disease (AD) (Li et al., 2025), Parkinson’s disease (PD), pain, amyotrophic lateral sclerosis (ALS), and brain trauma (Gardella and Moller, 2019; Menezes et al., 2020; Barbieri et al., 2023; Hebbar et al., 2024). Additionally, previous studies, as well as our latest research, have revealed that Nav1.6 plays an important role in the development and progression of tumors, including prostate, cervical, breast, and lung (both small cell and non-small cell) cancers, leukemias, and gliomas (Hernandez-Plata et al., 2012; Hernandez-Munoz et al., 2016; Lopez-Charcas et al., 2018; Lin et al., 2019; Li et al., 2022a; Ai et al., 2023). Therefore, integrating past and recent research on the relationship between Nav1.6 and related diseases, Nav1.6 is expected to represent a promising new direction for treatment. This paper summarizes the structure, expression, distribution, and electrophysiological characteristics of Nav1.6, particularly its relationship with clinically relevant diseases, based on the latest research, with the aim of providing a theoretical basis for intervention strategies for these diseases.

## Search Strategy

### Inclusion and exclusion criteria

**Figure 2:**
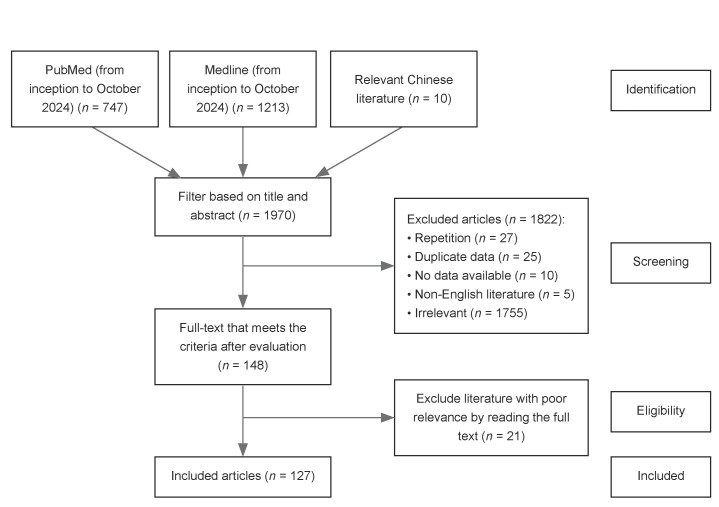
PRISMA flow diagram. PRISMA: Preferred reporting items for systematic reviews and meta-analyses.

A total of 127 articles (up to 2025) were included in this review (**Figure 2**), and the selection criteria are listed below.

**Inclusion criteria:** Any study investigating the pathogenesis, surveillance, diagnosis, and treatment of Nav1.6 and related diseases, including the associations between Nav1.6 and conditions such as epilepsy, AD, PD, pain, ALS, and traumatic brain injury (TBI), was included in this review. The experimental data were derived from human clinical studies, animal models (e.g., transgenic mice and rats), or cellular models (e.g., neurons and tumor cell lines). Non-English literature with English abstracts was included, and five Chinese articles were evaluated through full-text translation.

**Exclusion criteria:** Articles that mention only Nav1.6 but have no direct experimental evidence were excluded. Studies with unclear study methods or duplicate data were excluded.

### Literature search strategy

Literature searches were conducted in electronic databases such as PubMed using search terms including “nervous system,” “neurological diseases,” “VGSC,” “ AD,” “ PD,” “epilepsy,” and “Nav1.6.” Articles were selected based on their titles and content, with a focus on those published within the last 10 years; however, other references that met the inclusion criteria were also reviewed. Only articles published in English were considered.

### Weight analysis of animal models and clinical studies

Animal models (65%): The advantages of animal models include clear causality (e.g., sodium voltage-gated channel alpha subunit 8 (SCN8A) mutant mice validating the mechanism of epilepsy) and strong controllability. However, limitations include species differences (e.g., human Nav1.6 splice variants may be absent in mice), which increase the difficulty of fully mimicking complex diseases (e.g., cognitive decline in patients with AD).

Clinical studies (25%): The advantages of clinical studies include a direct association with human diseases (e.g., genotype–phenotype analysis of patients with SCN8A epilepsy). Limitations include small sample sizes (*n* < 50 in most studies) and the lack of long-term follow-up data.

Cell-based experiments (10%): These experiments are designed for mechanistic exploration and are often used in combination with *in vivo* studies.

### Effect of language bias on conclusions

This review primarily consists of English literature (95%) and may have overlooked important studies from non-English-speaking countries (e.g., the Chinese team’s report on the SCN8A epilepsy cohort has been supplemented by translation). Future research should systematically search multilingual databases (e.g., CNKI) and focus on integrating regional data. Current evidence supports Nav1.6 as a target for intervention in various diseases, but there is a need to address excessive extrapolation and language bias in animal models. In the future, it is essential to strengthen preclinical–clinical collaborative research and expand the coverage of non-English literature to enhance the generalizability of the conclusions.

## Structure, Expression, Distribution, and Electrophysiological Characteristics of Nav1.6

### Structure of Nav1.6

Nav1.6 was discovered by two separate research groups for the first time in the mid-1990s, approximately 10 years after the discovery of the Nav cDNA (Burgess et al., 1995; Schaller et al., 1995; Plummer et al., 1998). Among them, Burgess and colleagues discovered the mouse *Nav1.6* gene while cloning the genetic mutation responsible for motor end-plate disease in mice. They observed that this channel was abundantly expressed in both the brain and the spinal cord (Burgess et al., 1995). In parallel, Schaller and colleagues discovered the Nav1.6 cDNA in rat brains and reported the complete sequence of Nav1.6 in rats (Schaller et al., 1995). The gene SCN8A, encoding Nav1.6, was subsequently mapped to human chromosome 12q13 (Plummer et al., 1998).

As a tetrodotoxin (TTX)-sensitive VGSC, Nav1.6 shares higher sequence identities with other types of TTX-sensitive VGSCs than with TTX-resistant VGSCs. Specifically, the amino acid similarity of Nav1.6 to other VGSCs is as follows: Nav1.1 (77.6%), Nav1.2 (77.2%), Nav1.3 (76.1%), and Nav1.7 (72.4%) (Fan et al., 2023). Like other VGSCs, Nav1.6 consists of a core α subunit (with four homologous domains, each containing six transmembrane domains) and at least one auxiliary β subunit (1–4 auxiliary β subunits) (Catterall et al., 2005; Xu et al., 2019; Fan et al., 2023). The α subunit is the core component of the VGSC Nav1.6, with a high molecular weight (approximately 240–260 kDa). Structurally, the Nav1.6 channel comprises three primary components: four transmembrane domains (DI–DIV) with high sequence homology (> 75% amino acid similarity); three intracellular loops (two large loops, L1 and L2, and one smaller loop, L3); and the cytosolic N-terminal and C-terminal regions (N-T and C-T) (Catterall et al., 2005; Xu et al., 2019). Each transmembrane domain is composed of six hydrophobic α-helical segments (S1–S6). The transmembrane segment S4 contains 5–6 positively charged arginine residues, which are highly sensitive to changes in membrane potential and are referred to as the “voltage sensor” of VGSCs. This segment is closely related to the activation of VGSCs. The transmembrane segments S5 and S6 contain short SS1 and SS2 regions that cross the cell membrane in a “re-entrant” shape, known as the P-region. The P-region is located at the extracellular end of the sodium transmembrane transport chamber and is closely associated with the Na^+^ transmembrane transport process. The L3 loop connecting DIII and DIV features the characteristic isoleucine–phenylalanine–methionine (IFM) motif. This IFM structure facilitates rapid inactivation of VGSCs

In 2023, Fan et al. presented the cryo-election microscopy (cryo-EM) structure of human Nav1.6 for the first time. In their study, the cryo-EM structure of human Nav1.6 was identified in the presence of auxiliary subunits β1 and fibroblast growth factor homologous factor 2B at an overall resolution of 0.31 nm. The monolithic structure of Nav1.6 is an inactive state with a pore domain (PD) and all “up” voltage-sensing domains. The study demonstrated that conserved carbohydrate‒aromatic interactions involving tryptophan Trp302 and aspartic acid Asn326, as well as the β1 subunit, stabilized the extracellular loop in repeat domain I. In addition to the conventional lipids resolved via cryo-electron microscopy plots, the study revealed an unprecedented Y-shaped density that belongs to an unidentified molecule that binds to the PD (Fan et al., 2023). This finding provides a potential site for the development of Nav1.6-specific blockers, as well as a structural basis for further research on the relationship between Nav1.6 gene mutations and related neurological diseases, such as epilepsy.

### Expression and distribution of Nav1.6

Nav1.6 is broadly expressed in the nervous system and plays a key role in the electrophysiological activities of neurons, such as the initiation and propagation of APs in neurons (Caldwell et al., 2000; Hu et al., 2009; Lorincz and Nusser, 2010; Dumenieu et al., 2017; Zybura et al., 2021). In the central nervous system (CNS), Nav1.6 is significantly highly expressed in excitatory and inhibitory elements, such as pyramidal and granule cells of the hippocampus, cerebellar Purkinje neurons, pyramidal neurons, motor neurons, retinal ganglion cells, and granule cells of the hippocampus (Schaller et al., 1995; Osorio et al., 2005; Royeck et al., 2008). In addition, Nav1.6 can be expressed in many glial cells within the CNS and may play roles in phagocytosis, migration, proliferation, and the secretion of chemokines/cytokines (Schaller et al., 1995; Pappalardo et al., 2016; Sole and Tamkun, 2020). In the peripheral nervous system (PNS), Nav1.6 is expressed in various ganglion cells, including dorsal root ganglion (DRG) and trigeminal ganglion neurons, and its expression is essential for the transduction of peripheral sensory neurons (SNs) (Rush et al., 2005; Chen et al., 2018). Notably, Nav1.6 has also been detected in Schwann cells of the PNS, but its specific role in the function of Schwann cells is unknown (Schaller et al., 1995; Pappalardo et al., 2016).

In addition, previous studies have detected the distribution of Nav1.6 in neuronal substructures, including axons and dendrites. The specific distribution areas in axons are the axon initial segment (AIS) and the node of Ranvier, which play key roles in the generation and conduction of APs, respectively (Lorincz and Nusser, 2008; Kole and Stuart, 2012; Dumenieu et al., 2017; Huang and Rasband, 2018). More in-depth studies have shown that the expression level of Nav1.6 in the AIS of mature axons is approximately 30–80 times higher than that in the cell body (soma or proximal and distal dendrites), which mainly controls the initiation of APs distal to the axon initiation segment (Hu et al., 2009; Lorincz and Nusser, 2010). Nav1.2 contributes to the backpropagation of APs into cell bodies and dendrites (Burgess et al., 1995; Ai et al., 2023). Similarly, Nav1.6 proteins are widely distributed in dendrites, and their main role is to receive and integrate synaptic (information) afferents from neurons (Dumenieu et al., 2017).

**Figure 3:**
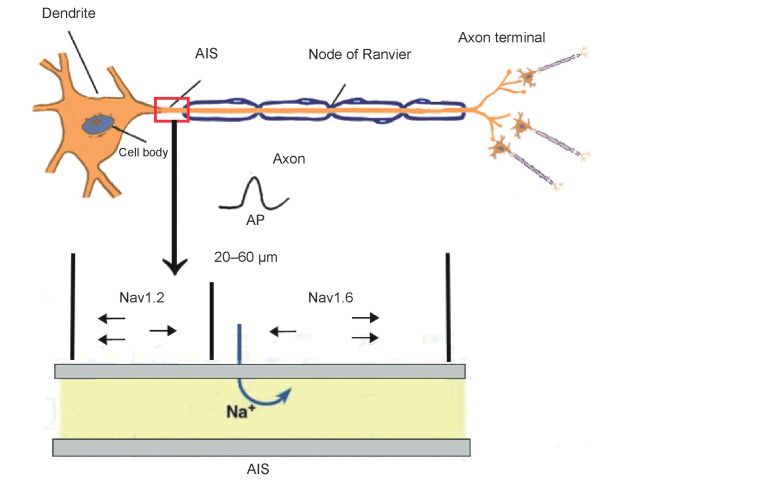
Differing contributions of Nav1.6 and Nav1.2 in action potential bursts and backpropagation. Modified from Yang et al. (2019). A high density of Na^+^ channels, mainly Nav1.2 channels and Nav1.6 channels, has been observed at the AIS of neurons. Action potentials erupt distally at the AIS. The Nav1.2 channels are clustered proximal to the AIS, promoting the backpropagation of action potentials to the cell body and dendrites, which is the basis for specific neuroplasticity. The Nav1.6 channels cluster at the distal end of the AIS and promote the burst of action potentials, which is the key to neuronal excitability. AIS: Axon initial segment; AP: action potential.

Differing contributions of Nav1.6 and Nav1.2 in AP bursts and backpropagation are shown in **Figure 3**.

### Electrophysiological analysis of Nav1.6 function

Nav1.6 has unique biophysical properties among VGSCs and has powerful neuronal signal transduction abilities. Studies have shown that Nav1.6 currents are deactivated more rapidly than those of other subtypes of VGSCs and show unique sodium ion currents, including continuous and resurgent currents (Raman et al., 1997; Smith et al., 1998).

In the brain and cerebellar neurons, the sustained current is generated mainly by Nav1.6, which produces an approximately fivefold higher current than that generated by Nav1.2 (Chen et al., 2008). Although these currents are usually small (0.5%–2.0% of the peak amplitude [Taddese and Bean, 2002]), electrophysiological studies have shown that sustained sodium currents can reduce the threshold for AP initiation and promote repetitive neuronal firing (Raman et al., 1997; Osorio et al., 2010). For example, cerebellar Purkinje neurons isolated from *SCN8A*-deficient mice have a 35% reduction in instantaneous sodium currents compared with those from wild-type mice, but they have a 70% reduction in continuous currents and reduced repeated discharges (Raman et al., 1997). In contrast, transgenic mice harboring mutations that increase persistent Nav1.6 sodium currents exhibit neuronal overexcitation, spontaneous epileptic activity, and even sudden, unexplained death (Veeramah et al., 2012; Patel et al., 2016; Lopez-Santiago et al., 2017). Therefore, the continuous current generated by Nav1.6 significantly affects the initiation and propagation of APs in synaptic transmission (Veeramah et al., 2012; Xie et al., 2013).

Nav1.6 also exhibits a unique resurrection current (Cummins et al., 2005). This distinct type of sodium current has voltage- and time-dependent characteristics, eliciting a small transient current after depolarization at an intermediate repolarization potential. Specifically, resurrection currents occur following depolarization and channel opening, during which a portion of the channel enters a blocked state that differs from and is faster than traditional fast inactivation. The phenomenon of resurrection currents was first observed in Purkinje neurons of the cerebellum (Raman et al., 1997; Raman and Bean, 1997), where they are believed to contribute to spontaneous electrical and polyphasic APs. In these studies, cultures from *SCN8A*-deficient mice showed a significant reduction in resurrection currents and an attenuation of repetitive AP discharges in cerebellar Purkinje neurons. The importance of resurrection currents in neurophysiology has also been demonstrated in model animals and electrophysiological studies (Rush et al., 2005; Barbosa et al., 2015; Patel et al., 2015, 2016; Pan and Cummins, 2020). These findings suggest that Nav1.6 produces a unique sodium current that is largely responsible for repetitive AP firing in neurons, indicating that the abnormal resurrection currents generated by Nav1.6 contribute to alterations in neuronal excitability. In addition to these unique sodium current characteristics, Nav1.6 exhibits rapid activation and rapid inactivation kinetics. Furthermore, Nav1.6 is known to show hyperpolarization shifts in terms of the voltage dependence of activation compared with other neuronal VGSCs (Smith et al., 1998; Rush et al., 2005), suggesting that Nav1.6 is activated earlier during depolarization. In cultured hippocampal neurons from *SCN8A*-deficient mice, a 60% to 75% reduction in persistent and recurrent currents was observed, along with a voltage-dependent depolarization shift of 5 mV in activation (Royeck et al., 2008). Additionally, neurons isolated from these mice exhibit an 8 mV depolarization shift at the spike threshold, resulting in decreased excitability. The activation threshold of the distal AIS, where Nav1.6 is concentrated, is hyperpolarized by approximately 12 mV (distal –55 mV, proximal –43 mV) compared with the proximal AIS near the cell body (Hu et al., 2009), which is the same as the effect of Nav1.6 on reducing the AP and initiation thresholds. In conclusion, the unique biophysical properties and subcellular localization of Nav1.6 provide flexible and complex determinants for the control of neuronal excitability.

### Signaling pathways and regulatory mechanisms of Nav1.6

VGSC-related disorders alter the expression and function of VGSCs, resulting in abnormal alterations in neurons. For example, the increased expression of sodium channels, plasma membrane trafficking, and altered permeability all contribute to the increased excitability of primary SNs. The regulation of sodium channel expression and function in these neurons is the substrate for protein kinases (protein kinase A [PKA], protein kinase C [PKC], and mitogen-activated protein kinase [MAPK]). Changes in the excitability of sodium channels are due to G protein-coupled receptor activation and downstream intracellular signaling pathways, which modulate sensory transduction and VGSCs present at the peripheral ends of these neurons (Cheng and Ji, 2008).

**Figure 4:**
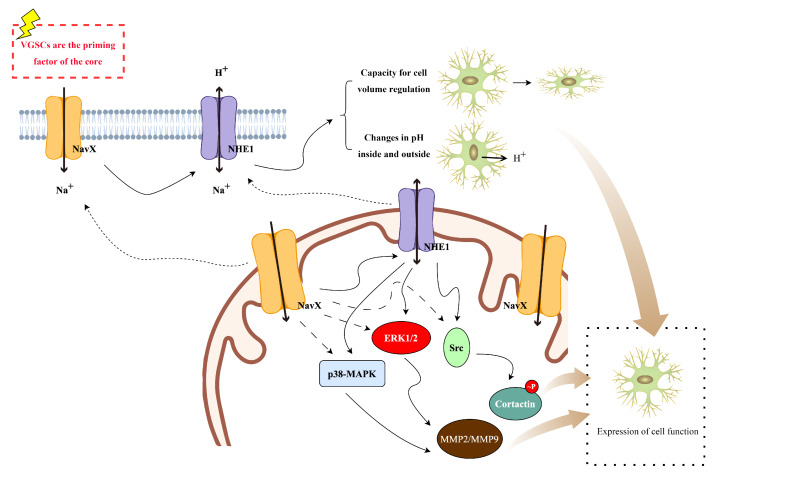
The role and mechanism of voltage-gated sodium channels. Multiple studies have proposed various mechanisms by which Nav1.6 regulates its activity. The function of Nav1.6 is associated with several signaling pathways that play key roles in nerve cells. Additionally, VGSCs also regulate cellular function by altering the pH of the cell. ERKs: Extracellular-regulated protein kinases; MMP: matrix metalloproteinase; p38-MAPK: p38 mitogen-activated protein kinase; pH: hydrogen ion concentration; VGSC: voltage-gated sodium channels.

The regulation of neuronal sodium currents by PKC and PKA occurs through phosphorylation at multiple sites on the channel. Peptide mapping and functional analyses of sodium currents have revealed four conserved serine residues—S554, S573, and S576 in the DI–VII linker, and S1506 in the DIII–DIV linker of Nav—as phosphorylation sites for PKA and PKC (Cantrell et al., 2002). PKA activation decreases the current resulting from the heterologous expression of Nav1.1, Nav1.6, and Nav1.8. Phosphorylation of S576 and S1506 by PKC promotes the phosphorylation of the DI-II loops by PKA (S554 and S573). In this way, the PKA and PKC pathways jointly regulate a single VGSC in a convergent manner. Modulating PKA/PKC-mediated peaks results in a decrease in the sodium current and slower kinetic inactivation, which may have important implications for neuronal AP thresholds and firing frequencies (Vijayaragavan et al., 2004; Chahine and O’Leary, 2014). The MAPK family consists of serine/threonine kinases that transmit signals from cell surface receptors to intracellular pathways. Dysfunction in MAPK signaling has been observed during stress and tissue injury and is associated with many neuronal disorders. The activation of p38 by MAPK (extracellular regulated protein kinase [ERK]1/2, p38) led to changes in the density of sodium currents at Nav1.6, but there were no changes in the gating characteristics of the sodium channels (Hudmon et al., 2008). Additionally, changes in the expression of splice variants of the sodium channel, induced by a sodium channelopathy, further modulate the ability of these signaling pathways to regulate the channel. In conclusion, deciphering the regulatory mechanisms of Nav1.6 may pave the way for innovative treatments for sodium channel-related diseases. **Figure 4** shows the mechanism of VGSCs.

## Nav1.6 and Related Diseases

### Nav1.6 and epilepsy

Epilepsy, which affects approximately 70 million people worldwide, is a common neurological disorder (Thijs et al., 2019). Due to its pathogenesis, it is considered a channelopathy (Catterall et al., 2010; Meisler et al., 2010). The relationship between VGSCs and epilepsy has been studied extensively. Mutations in VGSC-encoding genes, including Nav1.1/SCN1A, Nav1.2/SCN2A, Nav1.3/SCN3A, Nav1.6/SCN8A, and Nav1.7/SCN9A, have been shown to cause epilepsy (Menezes et al., 2020; Ademuwagun et al., 2021). Veeramah et al. (2012) first discovered pathological mutations in the *SCN8A* gene related to seizures in 2012. In the subsequent 10 years, hundreds of cases of epilepsy associated with mutations in the SCN8A gene were reported, and numerous relevant gene mutations have been identified (Gardella and Moller, 2019; Xie et al., 2019; Zaman et al., 2019; Menezes et al., 2020; Hu et al., 2022; Johannesen et al., 2022; Peng et al., 2022).

VGSCs may contribute to the development of epilepsy through several different mechanisms. The disruption of the balance between excitatory voltage-gated ion channels and inhibitory ion channels in the synaptic cleft due to the loss of function of inhibitory channels or enhancement of excitatory channel function may be the cause of some types of epilepsy. Another cause is a decrease in the threshold of ion channels involved in reducing the accuracy of spike timing, resulting in increased synchronicity, which can lead to painful symptoms. In addition, epileptic discharges regulate the level of sodium ions and calcium ions, which in turn promotes the excitation of neurons, forming positive feedback, which maintains epileptic activity (Debanne et al., 2024).

In 2022, Moller et al. conducted a detailed study involving 392 patients with neurological diseases (mainly epilepsy) caused by mutations in the *SCN8A* gene at multiple centers. The aim of this review was to investigate the clinical phenotype associated with the loss of function of the *SCN8A* gene. This study examined how changes in the characteristics of sodium currents affect neuronal firing behavior by simulating a model neuron with sodium, potassium, and leakage currents. Electrophysiological data were analyzed by evaluating the quantitative index score of the area under the curve of the resulting input–output curve. An analysis of clinical phenotypic, genotypic, and functional experimental evidence (electrophysiological data) revealed five main phenotypes of SCN8A-related diseases: benign familial infantile epilepsy, intermediate epilepsy, developmental and epileptic encephalopathy (DEE), generalized epilepsy, and neurodevelopmental disorder without epilepsy (Johannesen et al., 2022).

Moreover, researchers have analyzed the functional changes associated with seven Nav1.6 missense variants caused by *SCN8A* gene mutations. The results indicated that three missense variants [p.(Phe846Ser), p.(Leu840Pro), and p.(Asn1877Ser)] showed functional enhancement or caused increased neuronal firing; one allosteric variant [p.(Asn374Lys)] exhibited slight functional enhancement but did not alter the electrical activity of neurons; and the other three variants [p.(Val1758Ala), p.(Thr1787Pro), and p.(Ile1654Asn)] demonstrated a loss of neuronal function or decreased neuronal firing. Among the 170 individuals with known functional impairment, all 136 individuals with functionally enhanced variants had either focal epilepsy (*n* = 97, Groups 1–3) or unclassifiable epilepsy (*n* = 39), whereas the 34 individuals with loss-of-function (LOF) variants had generalized epilepsy (*n* = 14), no epilepsy (*n* = 11), or unclassifiable epilepsy (*n* = 6); only 3 patients had DEE. These results strongly suggest that benign familial infantile epilepsy, moderate epilepsy, and DEE are often caused by function-enhancing variants, whereas generalized epilepsy and neurodevelopmental disorders without epilepsy are associated with LOF variants. As the investigators reported, the clinical data from this study and the functional analysis of Nav1.6 following *SCN8A* gene mutations revealed a clear genotype-phenotype correlation between the age at seizure onset, seizure type, and the gain-of-function (GOF) or LOF effects of *SCN8A* gene mutations. Furthermore, a study has shown that the main epilepsy phenotype in patients with LOF allosteric variants is generalized epilepsy with absence seizures. The degree to which the electrophysiological function of GOF allosteric variants is disrupted is the primary determinant of the clinical severity of focal epilepsy. Pharmacological data from subsequent studies have suggested that sodium channel blockers, when used within 1 year of onset, are also a treatment option for *SCN8A* gene-associated focal epilepsy (Johannesen et al., 2022). Additionally, the application of a Nav1.6-selective blocker alone may represent a new treatment strategy for patients with Dravet syndrome, as the Nav1.6-selective blocker MV1312 has been shown to reduce the number of seizure events in Down syndrome animal models (Weuring et al., 2020). In addition, several new precise and promising Nav1.6-specific blocking drugs, such as XEN901, GS967 and Prax330, are being studied to provide an experimental basis for their clinical application (Baker et al., 2018; Bialer et al., 2018; Wengert et al., 2019).

A research team recruited 21 children (13 males and 8 females, two of whom were identical twins) carrying the new missense variant of SCN8A from three medical centers in southern China from January 2017 to February 2021. All 21 patients had *de novo* heterozygous mutations that were estimated to be pathogenic or probably pathogenic according to the American College of Medical Genetics and Genomics guidelines, with 2 and 14 mutations not previously reported. The study suggests that sodium channel blockers are the first choice of therapy for SCN8A epilepsy; however, responses to sodium channel blockers are closely related to clinical phenotype, genotype, and the function of the SCN8A missense variation. (Peng et al., 2022).

**Figure 5:**
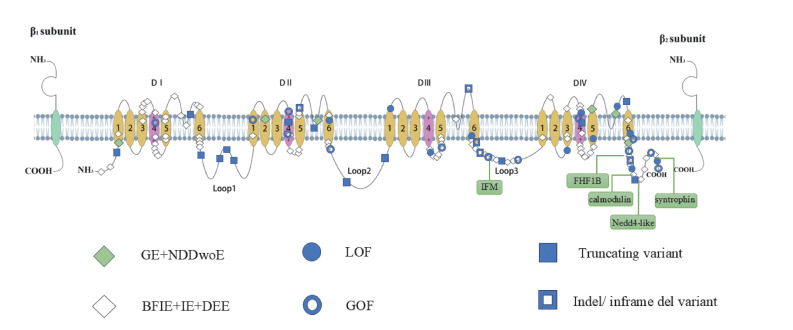
Schematic representation of the modulation sites in the voltage-gated Nav 1.6 sodium channel and the locations of variants associated with neurodevelopmental disorders. Modified from Wang et al. (2017) and Johannesen et al. (2022). The VGSC consists of a highly glycosylated pore-forming subunit (240–260 kDa) that functions independently, along with 1–4 auxiliary beta subunits (30.4–45 kDa). The schematic representation of the Nav1.6 channel highlights the locations of the pathogenic variants. Additionally, a schematic representation of five proteins reported to interact with VGSCs is provided. BFIE: Benign familial infantile epilepsy; DEE: developmental and epileptic encephalopathy; GE: generalized epilepsy; GOF: gain-of-function; IE: intermediate epilepsy; LOF: loss-of-function; NDDwoE: neurodevelopmental disorder without epilepsy; VGSCs: voltage-gated sodium channel.

Another retrospective study included a cohort of 50 Chinese patients with an *SCN8A* gene-related disease. The genetic and clinical characteristics of the patients and the treatment effects were further assessed. The pathogenicity of the variants was determined via next-generation sequencing. The 50 patients presented with severe DEE (70%), benign infantile epilepsy (12%), developmental encephalopathy with epilepsy (16%), and severe developmental delay without epilepsy (2%). The age at seizure onset ranged from 1 day to 23 months, and after antiepileptic therapy, seizures were controlled in 28% of patients; moreover, sodium channel blockers had a good effect on 26% of patients who had severe DEE (Hu et al., 2022). Schematic representation of the modulation sites in the voltage-gated Nav1.6 sodium channel and the locations of variants associated with neurodevelopmental disorders are shown in **Figure 5**.

In conclusion, the specific mechanism underlying the relationship between *SCN8A* gene mutations and seizures is still unclear. Studies have shown that in most patients with seizures caused by *SCN8A* gene mutations, the gene mutation results in Nav1.6 GOF and increased neuronal excitability. GOF mutants may be susceptible to sodium channel blockers (e.g., carbamazepine). However, some patients with SCN8A-associated epilepsy carry frameshift cessation or splice site mutations that cause LOF. This biophysical property of LOF results in a slight reduction in neuronal firing. This change is related to the inactivation of Nav1.6 due to abnormal functionality within the voltage sensor domain of DIV (Nakajima et al., 2019; Quinn et al., 2024).

However, the specific mechanisms by which different *SCN8A* gene mutations cause epilepsy need to be studied further because Nav1.6 is distributed mainly at the AIS, where the overexpression of Nav1.6 can lead to an increase in spontaneous and repetitive firing, thus leading to seizures.

### Nav1.6 and Alzheimer’s disease

AD is an irreversible and progressive heterogeneous neurodegenerative disease characterized by the gradual loss of neurons and synapses and amyloid plaque formation in neurons in the brain. In addition, the formation of intracellular neurofibrillary tangles composed of highly phosphorylated Tau filaments is a neuropathological hallmark of AD (Zhang et al., 2024). To date, the molecular mechanisms underlying the development of AD are not clear, and the specific physiological functions of key proteins, such as APP, presenilin 1 (PS1) and presenilin 2 (PS2), are also unclear (Zhang et al., 2024). Previous studies have shown that mutations in the APP gene are responsible for AD. Presenilin, which is involved in processing APP, is responsible for most familial AD cases involving mutations. Many different mutations in PS1 may be associated with hereditary early-onset AD (Gandy et al., 2001; Zhang et al., 2019). A recent study suggests that PS and APP are at the middle of a complex network involving many intracellular adaptogens, but the specific roles of these proteins in pathology or physiology are unclear (Ramazi et al., 2024).

Interestingly, many studies have indicated that the direct interaction of APP, its associated proteins, Aβ oligomers, and beta-secretase 1 (BACE1) enzymes with various VGSC isoforms may not only interfere with neurophysiological processes but also play a role in the onset and progression of AD (Liu et al., 2012, 2015; Li et al., 2016; Ciccone et al., 2019; Yuan et al., 2022; Barbieri et al., 2023; Baumgartner et al., 2023). As early as 2012, Busche et al. (2012) identified a correlation between AD pathophysiology, seizures, and increased neuronal excitability by studying a mouse model of AD. They reported that an increase in the number of overactive neurons was associated with the production of high levels of Aβ oligomers in the hippocampal CA1 region of young APP/PS1 mice. As a result, they proposed that soluble Aβ oligomers might directly cause neuronal hyperexcitability.

Nav1.6 has been a major subject of investigation regarding impaired neural activity in AD and its progression. For example, acute exposure to soluble Aβ oligomers can result in the overexcitability of CA1 pyramidal neurons (Ren et al., 2014). Moreover, selective increases in Nav1.6 expression and activity caused by soluble Aβ exposure have also been reported. Pyramidal cell hyperexcitability is abolished after treatment with VGSC blockers, suggesting that Nav1.6 is a driver of AD-associated increases in neuronal excitability (Ren et al., 2014; Ciccone et al., 2019). In addition, APP directly interacts with Nav1.6 and increases its surface expression through the G-coupled c-Jun N-terminal kinase (JNK) pathway (Liu et al., 2015; Li et al., 2016). This phenomenon enhances Nav1.6-mediated Na^+^ currents. These results strongly support a role for Nav1.6 in neuronal hyperexcitability during early AD and reveal novel, potentially synergistic mechanisms underlying AD pathogenesis (Li et al., 2016).

In 2015, Liu et al. reported that APP colocalizes and interacts with Nav1.6 in mouse cortical neurons. Knocking out APP reduces the Nav1.6 current amplitude and Nav1.6 cell membrane surface expression. Further studies revealed that the increase in the cell membrane expression of Nav1.6 induced by APP is Go protein dependent and is further promoted by constitutively active Go protein mutants and blocked by dominantly negative Go protein mutants. APP modulates JNK activity in a manner dependent on Go proteins, and suppressing JNK reduces the APP overexpression-induced upregulation of Nav1.6 cell membrane expression. Conversely, JNK also phosphorylates APP. The T668E mutation can increase the cell membrane expression of Nav1.6, the T668A mutation can reduce the cell membrane expression of Nav1.6, and the APP695 mutation mimics and prevents Thr668 phosphorylation. The phosphorylation of APP695 at Thr668 enhances its interaction with Nav1.6. Therefore, this study revealed that APP enhances the cell membrane expression of Nav1.6 through the Go protein-coupled JNK pathway. Li et al. (2016) subsequently investigated the interaction between APP and Nav1.6, and the results also suggested that APP regulates the Go protein-coupled JNK pathway through the APP-dependent phosphorylation of Nav1.6 at Thr668.

Amyloid-β (Aβ) precursor protein is central to the pathogenesis of AD (Hu et al., 2025; Kim et al., 2025; Zhang et al., 2025), but its physiological function is still not fully understood. In 2020, APP was also shown to be widely expressed in several interneuronal subtypes in the hippocampus and cortex. In a study of APP/APLP2 double knockout (cDKO) mice, behavioral deficits were associated with altered neuronal morphology, synaptic plasticity, and long-term potentiation. Impaired synaptic transmission in mice is associated with compound alterations in excitability/inhibitory synaptic currents and decreased CA1 pyramidal cell AP discharges, which together may contribute to hippocampal dysfunction (Mehr et al., 2020).

Interestingly, the study by Ciccone et al. (2019) revealed that the cause of abnormal neuronal activity in the hippocampal slices of 3-month-old Tg2576 mice is Nav1.6 overexpression, which suggests that Nav1.6 is a determinant of Aβ_1–42_ oligomer-induced hippocampal neuronal hyperexcitability. Therefore, selectively blocking Nav1.6 overexpression and/or hyperexcitability may be a new treatment option for counteracting hyperexcitability in the hippocampus and subsequent cognitive deficits in the early stages of AD.

The β-secretase enzyme BACE1 plays a role in the sequential proteolytic cleavage of APP and the development of plaques. The evidence suggests a correlation between BACE1 activity and the expression of sodium channels. Recently, a study of WT mice and APP/PS1 AD mice conducted by Yuan et al. (2022) revealed that Nav1.6 was overexpressed in aged AD model mice. In APP/PS1 mice, high Nav1.6 expression promoted BACE1 transcription through nuclear factor of activated T cells 1 (NFAT1) activation, which was dependent on NCX-mediated regulation (Na^+^/Ca^2+^ exchangers). Notably, the authors reported that the knockout of Nav1.6 via bilateral injection of an adeno-associated virus (serotype 8, AAV8) encoding an Nav1.6 shRNA into the hippocampus inhibited β-secretase-mediated APP cleavage, considerably reducing the density of Aβ plaques. As a result, cognitive deficits and neural network excitability were remarkably reduced. The authors further determined the molecular mechanism of Nav1.6 in the pathogenesis of AD. HEK293 cells overexpressing Nav1.6 were treated with TTX (a nonspecific blocker of CNS VGSCs), and a remarkable decrease in BACE1 expression was observed. When an shRNA was used to knock out Nav1.6, the TTX-induced reduction in BACE1 expression was less pronounced, revealing that this reduction was related to Na^+^ flux rather than the presence of VGSCs alone. Thus, Nav1.6 was identified for the first time as a target for slowing the progression of AD (Yuan et al., 2022).

The dynamic difference in Nav1.6 expression between early and late AD is worth considering. Age-matched sedentary APP/PS1 mice exhibited a gradual increase in cognitive and memory deficits with age. In sedentary APP/PS1 mice, Nav1.6 levels increased with age and differed considerably from those in age-matched WT mice. The change in Nav1.6 expression in AD model mice was reversed after 24 weeks of treadmill exercise, suggesting that treadmill exercise-induced improvements in neural activity and memory were associated with alterations in the number and distribution of hippocampal Nav1.6 in AD model mice (Tan et al., 2020).

Disease-modifying therapies for AD are still needed. Therefore, elucidating the neuronal mechanisms underlying AD progression is important for identifying novel interventions. Excessive hippocampal activity is one of the initial neurological changes observed in individuals with AD, contributing to both memory dysfunction and the progression of the disease. Therefore, correcting this hyperexcitability has been the focus of many recent studies (Ciccone et al., 2019; Yuan et al., 2022; Baumgartner et al., 2023). Although the roles of Nav1.1 and Nav1.6 in AD progression remain to be fully elucidated, the contributions of these channels to early-stage AD are being studied, and an understanding of these mechanisms may prove critical for the development of disease-modifying therapies for AD. The functional modulation of Nav1.1 and Nav1.6 is a promising therapeutic approach, considering the central roles of these channels in controlling the excitability of neuronal subtypes (Baumgartner et al., 2023).

In addition to conventional drug therapies, VGSC activity can also be modulated by regulating posttranslational modifications or stable interactions between VGSCs and accessory proteins that alter channel activity. The function and expression of numerous kinase signaling cascades and disease-associated proteins are altered in the brain during AD progression, some of which have functional effects on VGSCs. The functional outcomes of these interactions often vary among different Nav subtypes. Thus, modulating the function of VGSCs by altering the levels of the molecules that regulate their posttranslational modifications or associated protein complexes may aid in the development of therapies for different isoforms of specific VGSCs, improving their specificity for diseased tissues.

### Nav1.6 and Parkinson’s disease

PD is one of the most common age-related neurodegenerative diseases and is characterized by movement disorders such as stiffness, resting tremor, bradykinesia, and postural instability (Martinez and Peplow, 2025). These movement disorders are thought to be associated with the degeneration of the dopaminergic nigrostriatal pathway. The specific pathogenesis of PD is still unknown (Jovanovic et al., 2025). Biochemical studies of the pathogenesis of PD, studies of transplanted neurons in patients with PD, and studies of cell and animal models have shown that the abnormal aggregation of α-synuclein and the spread of pathology among the gut, brainstem, and higher brain regions may be the basis for the development of PD (Song and Kim, 2024). However, the role of sodium ion channels in the development of PD and the underlying mechanism are less reported (Morris et al., 2024).

**Figure 6:**
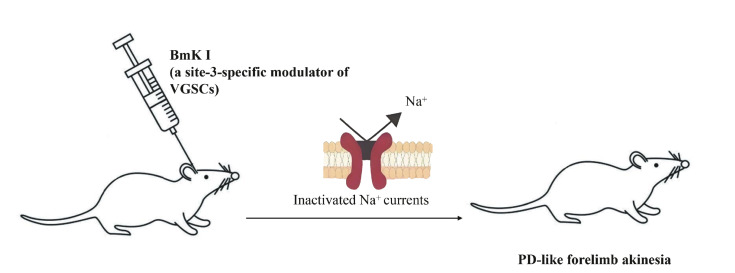
Animal models of PD were constructed using sodium channel blockers. BmK I, a toxin purified from the Chinese scorpion Buthus martensi Karsch that was identified as a 3-site-specific modulator of VGSCs, was injected into the GP of rats to induce forelimb akinesia. GP: Globus pallidus; PD: Parkinson’s disease; VGSCs: voltage-gated sodium channels.

Early studies revealed that VGSCs may play crucial roles in the abnormal electrical activity of globus pallidus (GP) and subthalamic nucleus (STN) neurons in patients with PD. For example, the scorpion toxin BmK I, derived from *Buthus martensi* Karsch, acts as a selective three-site modulator of VGSCs and was injected into the GP of rats to induce forelimb akinesia (Zhu et al., 2016). BmK I was unable to induce neuronal damage *in vivo* or *in vitro* 24 hours after treatment, suggesting that forelimb immobility was not caused by neuronal injury (**Figure 6**). An electrophysiological study further revealed that in human neuron-like SH-SY5Y cells, inactivated Na^+^ currents were more susceptible to regulation by BmK I than activated Na^+^ currents. Additionally, inactivation by BmK I was characterized by rapid inactivation, rather than slow inactivation. Therefore, BmK I may cause PD-like forelimb dyskinesia through the regulation of VGSCs in neurons. These results indicate that BmK I may be a valuable tool for studying the development of PD and further highlight the critical involvement of VGSCs in PD-related movement disorders.

In addition to motor symptoms, a variety of nonmotor symptoms are common in patients with PD. Among these, cognitive dysfunction is one of the most prevalent and significant. Unlike motor symptoms, the underlying mechanisms of cognitive impairment remain unclear. Motor symptoms may be associated with numerous factors related to PD pathogenesis, including damage from excess intracellular Ca^2+^ levels, mitochondrial dysfunction, oxidative or metabolic stress, and neurotoxins that increase the susceptibility of neurons to death. Abnormal neuronal firing in the GP and STN of PD patients is partly driven by VGSCs, which also play a role in cognitive decline (Zhu et al., 2016). After injecting 6-hydroxydopamine (6-OHDA) into rats to induce PD, Wang and colleagues reported that the expression of Nav1.1, Nav1.3, and Nav1.6 in the hippocampus increased dynamically at different time points following dopamine depletion (Wang et al., 2019). Compared with treatment with 6-OHDA, treatment with phenytoin (a sodium channel antagonist that retards the transition from the inactivated state to the resting state) significantly ameliorated cognitive impairment in rats (Wang et al., 2019).

Recently, several researchers have studied the role of Nav1.6 in the pathogenic mechanisms by which a unilateral 6-OHDA injection into the external GP causes motor deficits in rats (Liu et al., 2023). Nav1.6 expression was significantly increased in reactive astrocytes in the ipsilateral GP during the metaphase, but it was not altered in the later stages of the pathological process in 6-OHDA-injected rats compared to control rats. Additionally, the downregulation of Nav1.6 expression in the ipsilateral GP significantly ameliorated motor deficits in rats with 6-OHDA-induced lesions. Electrophysiological experiments revealed that this downregulation significantly reduced the abnormally high synchronization between the ipsilateral primary motor cortex (M1) and the GP in 6-OHDA-injured rats. Calcium imaging showed that suppressing Nav1.6 expression decreased the cytoplasmic calcium concentration ([Ca^2+^]_i_) in primary astrocyte cultures. These results indicate that elevated Nav1.6 levels in reactive astrocytes within the GP significantly contribute to motor impairments during the intermediate phase in 6-OHDA-lesioned rats, possibly by regulating [Ca^2+^]_i_ in astrocytes that communicate with neurons, thereby contributing to the generation of abnormal electrical signals in the basal ganglia of rats with 6-OHDA-induced lesions.

### Nav1.6 and amyotrophic lateral sclerosis

ALS is a fatal degenerative disease of the CNS characterized by the progressive neurodegeneration of motor neurons, and its etiology remains unknown (Feldman et al., 2022; Montiel-Troya et al., 2024). Despite extensive research, current treatments for ALS remain suboptimal from diagnosis to prognosis. A combination of pharmacological interventions, psychological support, and physical rehabilitation is most effective at enhancing the quality of life for patients with ALS. Among potential drug treatments, only a few are approved by the U.S. Food and Drug Administration (FDA) for the treatment of ALS and its symptoms. Other treatment modalities being considered include gene therapy, cell therapy, psychotherapy, physical therapy, and speech therapy, as well as robotics, alternative feeding methods, and communication devices (Hoxhaj et al., 2023). ALS leads to the progressive deterioration of the neuromuscular system, with death from paralysis typically occurring 3–5 years after symptoms first appear.

Currently, effective treatments that can slow or stop ALS-related neurodegeneration are unavailable (Hoxhaj et al., 2023). To date, two main drugs are used to treat ALS and are designed to prolong the patient’s life expectancy: riluzole and edaravone (Rokade et al., 2022). Although the mechanisms of action of these drugs are unknown, various hypotheses have been proposed. Riluzole is thought to regulate glutamate release and VGSC activity. In particular, this drug can decrease neuronal firing and inhibit VGSC production via sustained sodium currents.

Accumulating evidence suggests that a key pathophysiological event in the progression of ALS is hyperexcitability of the motor cortex (Menon et al., 2020). Increased circulating glutamate levels and hyperexcitability in the motor cortex of patients with ALS provide an empirical basis for the hypothesis of “dying forward” excitotoxicity. This hypothesis posits that increased activation of upper motor neurons propagates pathological changes to lower motor neurons in the spinal cord in the form of excess glutamate release, triggering an excitotoxic process. Many clinical trials have focused on therapies targeting excitotoxicity by inhibiting neuronal activation, but not all trials have been effective (Odierna et al., 2024). Although the molecular mechanism of ALS remains unclear, some evidence suggests that Nav1.6 may be a potential intervention target (Odierna et al., 2024).

Using G93A mice, Saba et al. (2019) found that the expression levels of Nav1.6 were altered in the primary motor cortex, with no expression observed in other cortical regions during ALS progression. This alteration was associated with changes in the excitability and sustained current in this region. Specifically, immunohistochemistry demonstrated that the expression of Nav1.6, a VGS primarily distributed in the CNS, was altered in the primary motor cortex, with no expression in other cortical regions of G93A mice during disease progression, as indicated by excitability levels and the persistent sodium current. Combined with electrophysiological recordings, microfluorometry experiments confirmed that hyperexcitation of the PNS resulted in greater accumulation of (intracellular calcium ([Ca^2+^]_i_) in P30 G93A mice compared with control mice. This difference persisted even when the PNSs of G93A and control mice generated APs at the same frequency. These results indicate that dysregulation of [Ca^2+^]_i_ in the PNS of G93A mice may lead to neuronal degeneration, highlighting Nav1.6 as a promising therapeutic avenue for slowing ALS progression (Saba et al., 2019).

In conclusion, these results suggest that motor cortex hyperexcitability is associated with ALS, suggesting that VGSCs may play an important role in cortical excitability, but the exact molecular mechanism underlying excitotoxicity deserves further investigation. Future studies are needed to elucidate the mechanism underlying the response to potential excitatory perturbations in the corticospinal tract induced by the pathological drivers of ALS at the neuronal network, cellular and synaptic levels.

### Nav1.6 and multiple sclerosis

Multiple sclerosis (MS) is a persistent autoimmune disease that damages the myelin sheath in the CNS and is accompanied by an influx of lymphocytes and macrophages (Haki et al., 2024). Similar to other conditions, MS is a degenerative CNS disease involving multiple factors, and its exact cause is still undetermined. Demyelinating plaques in the CNS accompanied by inflammation, gliosis, and neuronal degeneration are the pathologic hallmarks of MS. At the onset of the disease, lymphocytic infiltration that triggers axonal and myelin injury is reversible, but as repeated episodes of inflammation occur, microglial activation leads to extensive and chronic neuronal degeneration and ultimately disability.

Sodium channel blockers attenuate the inflammatory effects of microglial injury and activation, such as phagocytosis and the release of cytokines such as interleukin-1 and tumor necrosis factor-α (Black et al., 2009). Research has demonstrated that sodium (Na^+^) accumulation leads to the release of intracellular calcium (Ca^2+^). Elevated calcium levels activate nitric oxide synthase and harmful proteases and lipases, which contribute to axonal damage in patients with MS. This study suggests that partial blockade of VGSCs may provide neuroprotection, emerging as a potentially important strategy to prevent disability progression in MS patients (Yang et al., 2015).

**Figure 7:**
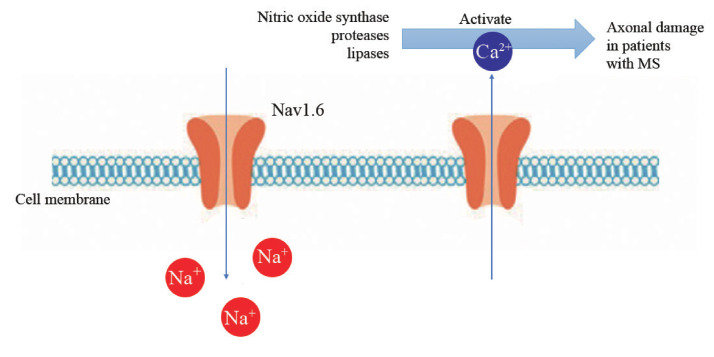
Possible mechanisms of Nav1.6 involvement in the pathogenesis of MS. Research has shown that sodium (Na^+^) accumulation leads to the release of intracellular calcium (Ca^2+^). Increased Ca^2+^ levels activate nitric oxide synthase and harmful proteases and lipases, which contribute to axonal damage in patients with MS. MS: Multiple sclerosis.

Similarly, experimental mouse models of autoimmune encephalomyelitis (EAE) have been used to study MS. In these mice, T cells infiltrate the CNS, initiating demyelination and leading to axonal loss (Hart et al., 2021). VGSCs contribute remarkably to axonal degeneration in MS. This study revealed that in demyelinated axons, the distribution of VGSCs is abnormal. Nav1.2 and Nav1.6 are present in plaques alongside NCX, whereas in myelinated control axons, Nav1.6 is located only in the nodes of Ranvier. Nav1.6-driven hyperactive Na^+^ currents force NCX exchangers into reverse mode, increasing intracellular Ca^2+^ concentrations in axons. The protease activation resulting from elevated Ca^2+^ concentrations leads to axonal degeneration. These results suggest that Nav1.6 may play an important role in the development of MS (Craner et al., 2004). Possible mechanisms of Nav1.6 involvement in the pathogenesis of MS are illustrated in **Figure 7**.

The above studies suggest that the VGSC Nav1.6 may play an important role in the occurrence and development of MS, especially in terms of axonal damage and neuronal degeneration (Yang et al., 2015; Hart et al., 2021), but its specific role and mechanism still need to be studied more systematically and in depth.

### Nav1.6 and pain

In addition to mediating APs, sodium channels contribute significantly to the propagation of pain signals in peripheral pain-sensing neurons. Many chronic pain syndromes arise from the overactivity of peripheral SNs. Sodium channels within these neurons play a key role in pain signaling. Most studies have shown that the activation of VGSCs, including the isoforms Nav1.3, Nav1.6, Nav1.7, Nav1.8, and Nav1.9, increases neuronal excitability and facilitates pain transmission in primary SNs (Zhao et al., 2025). Nav1.6 is widely expressed in both the peripheral and CNS and has been implicated in conditions such as epilepsy and migraine (Wagnon and Meisler, 2015). However, the role of these channels in human pain has not yet been clearly established. In the PNS, Nav1.6 is associated with trigeminal neuralgia, as it decreases the current threshold and increases the frequency of evoked APs in trigeminal ganglion neurons. Tanaka et al. (2015) first reported a GOF mutation of Nav1.6 associated with trigeminal neuralgia in 2016, supporting the assertion that Nav1.6 contributes to pain in humans. Nav1.6 is also one of the primary channels expressed in the DRG. In a rat model of neuropathic pain, local downregulation of Nav1.6 in the DRG effectively alleviated pain. Accumulating evidence suggests that Nav1.6 plays a significant role in chronic pain; however, it has not been identified as a factor in cancer-induced bone pain. Transcriptome sequencing has been performed to analyze transcriptional changes in the DRGs, revealing elevated Nav1.6 mRNA levels in rats following intratibial implantation of breast cancer cells. This suggests that p38 MAPK pathway-mediated activation of Nav1.6 expression in the rat DRG contributes to CIBP (Lin et al., 2022). This finding provides a potential avenue for mitigating cancer-induced bone pain.

Like all other cellular processes, the activation and inactivation of Nav1.6 are affected by temperature changes. The temperature-dependent gating of Nav1.6 has been studied and evaluated for possible specific regulation. A previous study revealed that cold and heat exacerbate the gating effect and sustained current of pain-associated Nav1.6 (Kriegeskorte et al., 2023).

One study showed that the CX3CL1/Nav1.6 pathway plays a key role in the transformation of acute pain into chronic pain caused by neuroinflammation in the anterior cingulate cortex (Li et al., 2022b). Inflammatory cytokines play important roles in the establishment and maintenance of chronic pain (Liu et al., 2017). Tumor necrosis factor-alpha (TNF-α) is a cytokine that is prominently involved in the pathogenesis of neuropathic pain. A study revealed the functional significance and role of VGSCs and inflammatory cytokines in the pathogenesis of neuropathic pain. The application of the cytokine TNF-α increases sodium channel currents in DRG neurons (Leo et al., 2015). TNF-α increases the expression of Nav1.6 in DRGs.

In addition to drug therapy, Ladez et al. (2024) investigated the mechanism by which VGSCs affect pain in the treatment of chronic pain involving the DRG. The process was computationally simulated in a neuron simulation environment. DRG stimulation resulted in a remarkable increase in the sodium concentration and a decrease in the potassium concentration within axons, which together disrupted the transaxonal ion gradient. This disruption resulted in activity-dependent conduction slowing, resulting in the eventual blockage of Aδ and C-fiber afferent nerves, which relieved pain.

Nav1.6 is a key target for pain management, but the specific mechanism of its upstream regulation remains unclear. At present, further studies of the underlying mechanism are urgently needed to better develop drugs for pain relief from the perspective of Nav1.6.

### Nav1.6 and brain trauma

**Figure 8:**
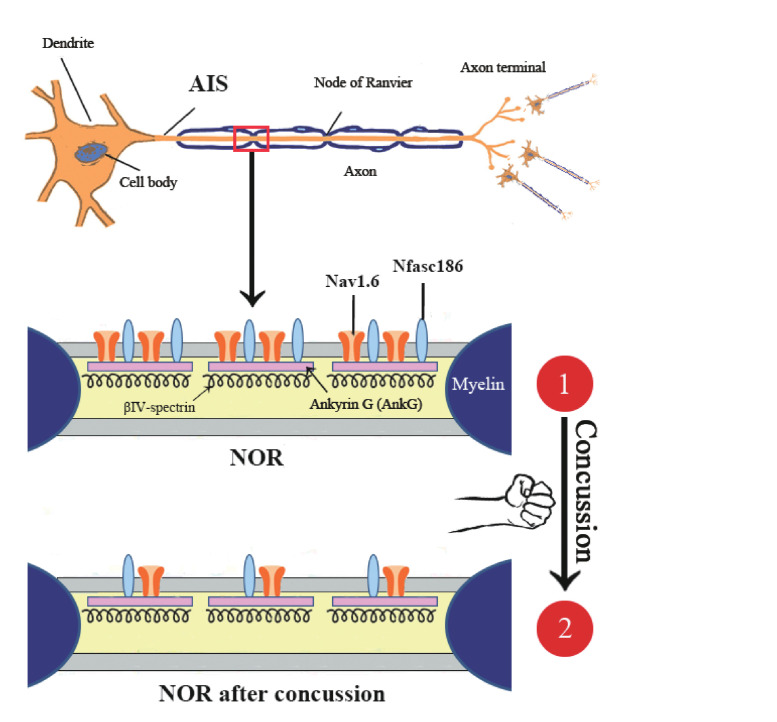
The changes in Nav1.6 after the experimental shock. The image shows the changes in Nav1.6 after the experimental shock. (1) Before the concussion, Nav1.6 and proteins (including βIV-spectrin, AnkG, and Nfasc186) were enriched in the NOR area. (Light yellow, myelin; purple, myelin; orange, Nav1.6; pink, AnkG; black, βIV-spectrin; light blue, Nfasc186). (2) However, after concussion, the expression of Nav1.6 in the NOR decreased, accompanied by a remarkable deletion of Nfasc186. In conclusion, these experimental concussions led to the gradual loss of Nav1.6 and alteration of the NOR cytoskeletal scaffold to form a type of sodium channel disease. AIS: Axon initial segment; NOR: node of Ranvier.

An estimated 50–60 million cases of TBI occur each year internationally. Approximately 90% of these cases are considered “mild” TBIs or concussions (Nelson et al., 2019). Despite the high incidence of concussions, the pathophysiology of concussions is poorly understood. A new type of sodium channelopathy has been found to be an important pathology of concussion (Song et al., 2022). Song et al. (2022) used a head rotational acceleration pig model scaled with injury parameters with human concussion biomechanics that shows DAI under pathology without features of moderate or severe TBI. Evolutionary changes in node of Ranvier cytoskeletal VGSC expression and the morphological integrity of myeloid axons were then assessed for changes similar to those observed in injured axons from patients with TBI. These results suggest that damage-induced changes in VGSCs may play a key role in disrupting the brain network signaling present in DAI (Song et al., 2022). **Figure 8** shows the changes in Nav 1.6 after the experimental shock.

The aggregation and distribution of Nav1.6 in the NOR is largely determined by its binding to βIV spectrin, as well as its interaction with the cytoskeletal complexes of ankyrin G and neurofascin-186 (Nfasc186). After an experimental concussion, the expression of Nav1.6 within NOR is reduced, along with a remarkable loss of Nfasc186. Experimental concussion leading to progressive loss of Nav1.6 is a manifestation of this sodium channelopathy (Song et al., 2022).

Currently, physical rehabilitation remains the only established treatment for TBI. Research indicates that VGSCs are significantly upregulated following TBI and play a key role in driving its pathological progression (Denomme et al., 2020). Very early after TBI, the messenger ribonucleic acid and protein expression of Nav1.3 and Nav1.6 were significantly upregulated in the ipsilateral injured cortex of rats. This upregulation is related to the severity of TBI and is associated with diffuse axonal damage. Mechanical damage to axons results in the influx of sodium through VGSCs, which subsequently triggers calcium influx, leading to neuronal death. Exercise-induced cognitive improvement has been associated with sodium channel-mediated excitability (Trompoukis and Papatheodoropoulos, 2020). In subsequent studies, mice received voluntary running wheel exercise therapy prior to the injury and/or for 3 weeks after the injury, as well as combination therapy before and after TBI. Behavioral changes, electroencephalogram (EEG) recordings, and VGSC expression levels in the mice were then assessed. The impact of cell viability on exercise-induced injury in mice can be evaluated by examining neuronal hyperexcitability, sodium current alterations, and the protein expression of VGSCs in neurons damaged by TBI (Wang et al., 2024). Therefore, interventions related to sodium channels may be considered as options for the adjuvant treatment of TBI in the future.

## Clinical Translational Research

In 2023, Ai et al. screened potential drugs for Nav1.6-related treatments through virtual screening and drug susceptibility analysis. TargetMol was used to identify a library of FDA-approved and pharmacopeia-indexed molecules, which included 2858 drugs and their structures. In this study, results from clinical trials and FDA-approved drugs were selected, and virtual screening and molecular docking of FDA-approved drugs were performed based on the three-dimensional structures of proteins. The study screened the ten drug molecules with the highest scores. A molecular docking and binding interaction study of SCN8A with the drugs revealed that neomycin sulfate B (T2096), paromomycin sulfate (T1104), neomycin sulfate (T0950), plerixafor 8HCl (T1776 and T1776L), and isavuconazonium sulfate (T3934) exhibited strong binding affinity to SCN8A. The results of the NCI-60 drug susceptibility analysis also revealed that drug concentration and sensitivity to three drugs (tamoxifen, selumetinib, and tepotinib) were significantly correlated with the expression of SCN8A, and variations in SCN8A expression levels influenced the IC50 values of the drugs. Many of these screened Nav1.6-related drugs have demonstrated therapeutic value in both non-oncological and oncological diseases (Ai et al., 2023). Using the AlphaFold protein structure database, we obtained the 3D structure of SCN8A. The GHECOM algorithm was employed to identify potential binding sites, followed by virtual screening and molecular docking simulations performed with UCSF DOCK 6.9. Finally, the docking conformation was determined using PyMOL, and the interaction forces were analyzed using LigPlus. Separate Pearson correlation analyses were conducted to assess the relationship between the expression of each gene and different drugs, and the results from clinical trials were compared with those for FDA-approved drugs. The results of the analysis were filtered according to a *P* value of less than 0.05. After the analysis, virtual screening and molecular docking for FDA-approved drugs were performed based on the three-dimensional structure of the protein. The docking conformation and interaction analysis of SCN8A with the drugs indicated that neomycin sulfate B (T2096), paromomycin sulfate (T1104), neomycin sulfate (T0950), plerixafor 8HCl (T1776 and T1776L), and isavuconazonium sulfate (T3934) exhibited strong binding forces (**Additional Table 1**).

**Additional Table 1:** Six FDA-approved drug molecules with high docking scores and strong binding affinity to the SCN8A protein

ID	Drug	Grid score
T2096	Neomycin sulphate B	-122.5961
T1104	Paromomycin sulfate	-122.1724
T0950	Neomycin sulfate	-122.1169
T1776	Plerixafor 8HCl	-121.5208
T1776L	Plerixafor 8HCl	-120.1207
T3934	Isavuconazonium sulfate	-112.7299

Modified from Ai et al. (2023). FDA: Food and Drug Administration; GHECOM: geometry-based hole and cavity estimation and optimization method; UCSF DOCK: University of California, San Francisco DOCK; PyMOL: python molecular viewer; LigPlus: ligand interaction diagrams plus; SCN8A: sodium voltage-gated channel alpha subunit 8.

Currently, Nav1.6-targeted drugs are primarily being developed for epilepsy and neuropathic pain, with some having entered clinical trials.

In 2022, a Nav1.6 inhibitor called XEN-901 (NBI-921352) was introduced. This drug preferentially binds to open and inactive state channels to reduce neuronal hyperexcitability. It is currently undergoing clinical trials for epileptic encephalopathy and focal epilepsy. However, this drug must balance CNS permeability with cardiac safety concerns (Johnson et al., 2022; Müller et al., 2024).

Topiramate is a nonselective sodium channel inhibitor, but it selectively inhibits INaPs in Nav1.6, including the N1466T/K mutation. While topiramate is effective in some patients with Nav1.6 mutation-associated epilepsy, it lacks subtype specificity (Guo et al., 2022). BmK IT2, a β-scorpion toxin derived from scorpion venom, selectively inhibits Nav1.6-mediated sodium currents without affecting Nav1.7 or Nav1.8. This drug is currently being studied in preclinical trials and is expected to be used for refractory epilepsy (Feng et al., 2015).

Research conducted by the Shanghai Institute of Materia Medica, Chinese Academy of Sciences, revealed that NK728, a small-molecule Nav1.6 inhibitor, exhibited antiepileptic activity in the maximal electroconvulsive shock model (Guo et al., 2022). The Huang Zhuo team from Peking University proposed that a Slack–Nav1.6 coupling modulator could interfere with the interaction between Slack potassium channels and Nav1.6, thereby enhancing the antiepileptic effect of quinidine (with IC50 reduced by 100-fold). These studies represent explorations of Nav1.6-related drugs for the treatment of epilepsy (Yuan et al., 2024). Similarly, Nav1.6 has been extensively studied in the context of neuropathic pain. The downregulation of Nav1.6 expression by miR-30b has been found to alleviate oxaliplatin-induced neuralgia, as validated in animal models (Li et al., 2019). These findings provide new avenues for gene therapy or the development of miRNA mimics.

The regulatory mechanisms of Nav1.6 in different diseases vary, and drug design must consider factors such as subtype selectivity, state dependence, and tissue specificity (**Additional Table 2**). The future of drug design should be precise and efficient, and the analysis of isoform-selective blockade must be emphasized. Nav1.6 is structurally similar to other isoforms, necessitating the design of allosteric site inhibitors based on the Nav1.6-β1-FHF2B complex resolved by Yan Ning’s team using cryo-EM (Fan et al., 2023). BmK IT2 achieves selectivity by binding to the voltage-sensing domain of Nav1.6 (Feng et al., 2015). Genetic intervention is also expected to be a prominent topic in the future. For example, intrathecal injection of miR-30b analogs has been shown to reduce the expression of Nav1.6 in the DRG to treat pathological pain (Li et al., 2019). Importantly, peripheral modification of drugs is necessary, such as increasing the polarity of the drug to reduce penetration of the blood–brain barrier and minimize side effects.

**Additional Table 2:** Nav1.6-related drug design approaches for different diseases

Disease	Nav1.6 modulation	Potential drug strategy	Representative drug/method
Epilepsy	Enhancements	Pore blockers Allosteric adjustment	XEN-901, BmK IT2, and NK72813 (Feng et al., 2015)
KCNT1-associated epilepsy	Slack-Nav1.6 coupling enhances neuronal excitability	Interference channel coupling	Quinidine + Nav1.6 inhibitors (Yuan et al., 2024)
Neuropathic pain	Increased expression of Nav1.6 in DRG neurons	miRNA regulation, MAPK/ERK pathway inhibition	miR-30b analogs, U0126 (ERK inhibitor) (Li et al., 2019; Shao et al., 2023)
Perioperative neurocognitive impairment	Increased hippocampal Nav1.6 expression	State-dependent blockade	Lidocaine (low-dose CNS delivery) (Xia, 2023)

In recent years, important progress has been made in the development of drugs targeting Nav1.6. At present, Nav1.6- targeted drugs are mainly used for epilepsy and neuropathic pain, and some have entered clinical trials. DRG: Dorsal root ganglion; KCNT1: potassium channel subfamily T member 1; MAPK/ERK: mitogen-activated protein kinase/extracellular-regulated protein kinase.

In conclusion, Nav1.6 is a key target for neurological diseases, and the development of Nav1.6-targeted drugs is shifting from nonselective blockade to precise strategies such as isotype-specific blockade, allosteric regulation, and gene intervention. In the future, a combination of structural biology, single-cell sequencing (such as profiling Nav1.6 expression in neuronal subsets), and clinical translational research is needed to drive breakthroughs in next-generation Nav1.6-targeted therapies.

## Limitations

Most of the studies included in the literature are prospective studies, which may have caused selection bias. This review only searched for studies published in English, and studies published other languages were not included. The results of studies published in other languages need to be further examined. Many types of sodium ion channels exist, the mechanism of each subtype is different, and the current research does not fully cover Nav1.6, which reduces the comprehensiveness of the research results. **Additional Table 3** evaluates the cutting-edge technologies and methods used in current research of Nav1.6 and compares them in three dimensions: technical advantages, limitations, and applications. The purpose of this table is to provide researchers with a reference basis for selecting technology.

**Additional Table 3:** Comparative analysis of new technologies and methods of Nav1.6

Technique/Method	Advantage	Limitation	Application example
Cryo-EM	Atomic-level resolution (0.31 nm)	Large quantities of protein are purified	Analysis of the structure of the Nav1.6-β1-FHF2B complex
Single-cell sequencing	Reveals neuronal subset- specific expression	Unable to directly correlate with electrophysiological functions	Discovery of high expression of Nav1.6 and sustained currents in Purkinje cells
Virtual drug design	Rapid screening of allosteric regulatory sites	Rely on existing structural data	Prediction of conjugated drugs
Animal organoid models	Simulate the human disease microenvironment	The cost is high and the cultivation cycle is long	Test the effect of Nav1.6 inhibitors on reversing AD neuron hyperexcitability

AD: Alzheimer's disease; Cryo-EM: cryo-electron microscopy; FHF2B: fibroblast growth factor homologous factor 2B.

## Conclusions and Prospects

**Figure 9:**
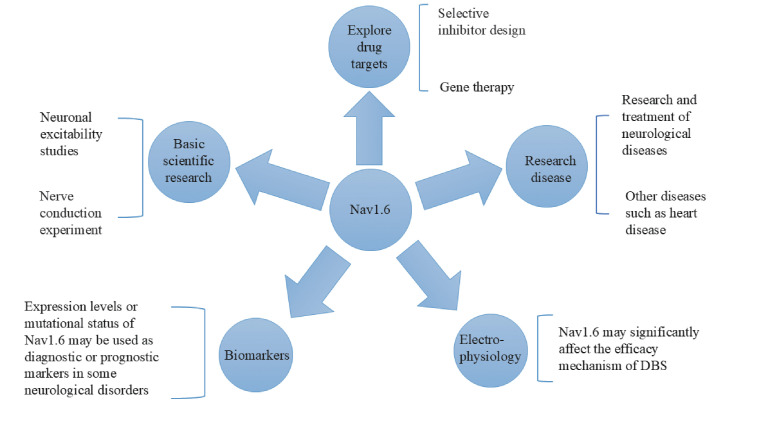
Prospects and application areas of Nav1.6. Based on the current research progress, we summarize the specific research directions in the figure. With the development of new technologies and the cross-integration of multiple disciplines, Nav1.6 research is expected to make breakthroughs in the future, leading to revolutionary changes in the diagnosis and treatment of related diseases. DBS: Deep brain stimulation.

The role of Nav1.6, a key subtype of VGSCs, in neurological diseases has received increasing attention, but many key questions remain to be explored (**Figure 9**). In the future, single-cell RNA sequencing and spatial transcriptomic technology can be used to systematically map the expression profile of Nav1.6 in neuronal subsets (e.g., excitatory/inhibitory neurons) and glial cells in different brain regions. Combined with patch–clamp technology, a quantitative model of the relationship between the Nav1.6 expression level and neuronal electrophysiological characteristics was established. Precise methods for drug development, such as a cryo-EM-based analysis of the Nav1.6 structure, can further optimize highly selective inhibitors (e.g., XEN901 and GS967) and explore allosteric modulation strategies to reduce interference with normal neurological function. Differentiated regulatory approaches are needed for different diseases (e.g., GOF mutations in epilepsy). In-depth research on disease mechanisms and biomarkers and investigations of how Nav1.6 interacts with pathological proteins such as Aβ and α-synuclein in neurodegenerative diseases such as AD and PD are needed. The potential of Nav1.6 expression levels or mutation profiles as markers for early diagnosis or prognosis of disease should be explored. The combination of gene therapy and precision medicine could be developed. For example, for epilepsy caused by *SCN8A* mutations, gene editing/regulation techniques such as clustered regularly interspaced short palindromic repeats or antisense oligonucleotides can be explored. Treatment should be individualized based on the patient’s genotype (e.g., GOF *vs*. LOF mutations). More importantly, a need exists to address the off-target effects of Nav1.6 subtype-selective drugs, as well as blood–brain barrier penetration. The combination of basic research and clinical translation should be strengthened, and the entry of existing drug candidates into clinical trials should be promoted. Nav1.6 research has broad prospects in elucidating disease mechanisms and developing targeted therapy, but multidisciplinary collaboration is needed to overcome the technical bottlenecks and finally achieve the leap from the laboratory to the clinic.

Nav1.6 is a key regulator of neuronal excitability, and studies of its structure, distribution, and electrophysiological properties have established its multifunctional role in neurological diseases and tumors. Nav1.6 is widely distributed in the CNS (including the hippocampus, cortex, and cerebellum) and the PNS (such as in the dorsal root ganglia), especially in the AIS and nodes of Ranvier, which are involved in the initiation and conduction of APs. Nav1.6 generates sustained and resurgent currents, considerably lowers the AP threshold, and promotes the repetitive firing of neurons. Its rapid activation and inactivation properties play a key regulatory role in neuronal excitability. Dysfunction of Nav1.6 is implicated in the pathogenesis of epilepsy, AD, PD, ALS, and other conditions by affecting neuronal excitability, signaling pathways (such as the c-Jun N-terminal kinase and Janus kinase-signal transducer and activator of transcription pathways), and ion balance. Epilepsy: Mutations in the *SCN8A* gene result in either a gain (GOF) or loss (LOF) of Nav1.6 function and are associated with multiple epilepsy subtypes. GOF mutations cause focal epilepsy, while LOF mutations are linked to generalized epilepsy. Nav1.6-selective blockers (e.g., XEN901 and GS967) have shown therapeutic potential. AD: Nav1.6 plays a crucial role in neuronal hyperexcitability induced by Aβ oligomers in the early stages of AD. APP increases Nav1.6 membrane expression through the Go-JNK pathway, while BACE1 transcription is regulated by Nav1.6. Inhibition of Nav1.6 reduces Aβ plaque formation. PD: Nav1.6 is upregulated in astrocytes in PD models and is involved in dyspraxia and abnormal neuronal synchrony. Sodium channel blockers, such as phenytoin, may improve cognitive impairments. ALS: Nav1.6 contributes to motor cortex hyperexcitability and may be associated with neuronal death due to calcium dysregulation. Riluzole delays ALS progression by modulating sodium channel activity. MS: The abnormal distribution of Nav1.6 in demyelinating axons leads to a reversal of sodium/calcium exchangers, resulting in calcium-dependent axon injury. Sodium channel blockers have neuroprotective potential. Drugs targeting Nav1.6 (e.g., specific blockers) have shown promise in treating epilepsy, AD, and tumors, but their selectivity and safety need further optimization. Future research should focus on exploring the molecular mechanisms of Nav1.6 in various diseases, developing subtype-specific regulatory strategies, and expanding clinical translational research. Strengthening preclinical–clinical translational research is necessary to clarify the dynamic role of Nav1.6 in different disease stages and to optimize the spatiotemporal specificity of targeted therapies.

In conclusion, the VGSC Nav1.6 may play an important role in the occurrence and development of neurological diseases and may play different roles in different types of neurological diseases. For example, Nav1.6 plays a crucial role in the initiation and propagation of APs during pain signaling. Studies on the use of electrical stimulation to treat pain have been reported. Mutations in *SCN8A* at the ALS site lead to the development of epilepsy. Multiple studies have proposed multiple mechanisms by which Nav1.6 regulates its activity. The function of Nav1.6 is related to several signaling pathways that play key roles in nerve cells. Therefore, targeting Nav1.6 using small-molecule antagonists or genetic engineering to modulate its function and expression is a promising approach. The specific mechanism of Nav1.6 still needs to be further studied in the future to reveal the specific relationship between sodium channels and the occurrence and development of neurological diseases, which will be a hot topic.

## Additional files:

***Additional Table 1:*** *Six FDA-approved drug molecules with high docking scores and strong binding affinity to the SCN8A protein.*

***Additional Table 2:*** *Nav1.6-related drug design approaches for different diseases.*

***Additional Table 3:*** *Comparative analysis of new technologies and methods of Nav1.6.*
